# Editorial: Rising ideas in: theoretical and philosophical psychology

**DOI:** 10.3389/fpsyg.2023.1269309

**Published:** 2023-09-26

**Authors:** Chiara Fini, Fausto Caruana, Anna M. Borghi

**Affiliations:** ^1^Department of Dynamic and Clinical Psychology, and Health Studies, Sapienza University of Rome, Rome, Italy; ^2^Institute of Neuroscience of the National Research Council (CNR), Parma, Italy; ^3^Institute of Cognitive Sciences and Technologies, Italian National Research Council, Rome, Italy

**Keywords:** epistemology, ethics, artificial intelligence, enactivism, numerical communication

This Research Topic was aimed at collecting high-quality work of international researchers on novel ideas in theoretical and philosophical psychology. The contributions are highly interdisciplinary, encompassing cognitive linguistics, psychology, neuroscience, psychoanalysis and Artificial Intelligence, and are all characterized by a solid theoretical background.

The editorial touches upon the intertwined relationship between mind and body. Particular attention is drawn to the epistemological issue and the role of the body in research practice and creativity. Another raised issue encompasses the impact of AI research on the psychological research field. A final issue taps into the challenges and new methodological approaches to studying cognition in psychological research.

Regarding the mind-body theme, Meyer and Brancazio focus on enactivism and on how it is conceived. The authors argue that enactivism does not represent a real alternative paradigm to cognitivism. Cognitivism is not looming in a crisis, hence enactivism cannot be seen as a revolution or a paradigm shift in a strong, Kuhnian sense, or as a more attractive alternative to cognitivism in understanding cognition. Rather than a scientific program aimed to replace cognitivism, the authors propose to conceive enactivism in broader terms as a philosophy of nature, able to integrate interdisciplinary research programs within a comprehensive and coherent view of mind and life.

From a completely different perspective, Zhang et al. offer a theoretical reflection on two classical conceptualizations of mind-body relationship: Merleau Ponty's phenomenological one and Freud's psychoanalytic one. For Merleau Ponty, the unconscious consists of all of the content outside of consciousness, including desire, feelings, emotions, and some unconscious concepts. For the phenomenologist, indeed, the content of the unconscious mind is experiential, the point where mind and body meet, it is more open and permeable, or an “echo of others in me, of me in others.” The dynamic structure of the unconscious for Merleau Ponty resides in the body and in psychological activities, including somatic sources. While for Merleau-Ponty, the unconscious cannot be conceptualized as the boundary between mind and body, Freud looks at the unconscious as derived from the libido of the human body and as the boundary between body and mind. In Freud's view, the unconscious was latent and susceptible to be accessed by overcoming resistance, through the psychoanalytic method. It could not be acquired through conscious reflection, but only through the analysis of many phenomena in conscious life, including verbal errors, associations, dreams, and actions. Unconscious for Freud can also be thought also the “cradle” of human creativity.

This latter, following the socio-cultural and 4E “embodied, enacted, embedded, extended” cognition perspectives (Newen, 2018), serves the function of guiding actions, as proposed in their manuscript by Gubenko and Houssemand. The authors describe the Alternative Uses Task (AUT), which focuses primarily on associative and divergent-convergent accounts, i.e., on the idea that creativity involves the ability to retrieve and connect distant concepts, to generate original ideas, but also to select them in relation to current standards and conventions. This view might be seen as “disembodied” since it does not sufficiently explain how ideas are translated into action. In the AUT, participants typically have to engage with objects not present in the environment. The authors outline an embodied alternative to AUT, in which children are invited to find different uses for a real object, a paper cup, allowing them to explore its affordances. They propose that new strategies for action in adults are similar to those adopted in children's pretend play, and are the result of the combination of sensorimotor experiences derived from object affordances and sociocultural experiences linked to canonical object use. In this framework, they emphasize the role of language, which can be used as a tool to create new categories and design new action possibilities. Linguistic production and human creativity are nowadays undergoing the impact of a new powerful tool: artificial intelligence (AI).

In this regard, El Maouch and Jin provide an exhaustive reflection on the theoretical and methodological aspects of artificial intelligence and its interaction with the psychological field. They argue that the historical body-mind dichotomy, which has always characterized the philosophy of mind, also affects the theoretical background of AI. Specifically, they identify some weaknesses in the theoretical background of AI: the absence of a unified object of study, artificial intelligence as an empirical tool embracing multiple domains of knowledge and application; the absence of theoretical worldview scaffolding; the denial of theoretical crisis because of the empirical success of AI; the eclecticism and theoretical proliferation. The authors then, by capitalizing on the cultural-historical activity theory (CHAT), and on Vygotsky (1962) dialectical logic, advance the idea that processes of symbolization and abstraction develop from contradiction-based meaning. The Concatenation of events, interpreted as the absence or presence of output, represents the ground of learning processes. By grasping formal contradictions, AI can abstract, generalize and symbolize. The agent does not need content-based static knowledge acquired through the physical experience, as it is endowed with methodologies as a source of knowledge.

The issue of AI opens inevitably room for ethical debates: more than others its implications and effects on education. Yu and Yu, in their systematic review, deal with the issue of data privacy during online learning, since AI systems can easily access information on the learners and their learning strategies, which might generate prejudice and influence the teachers; in a similar way, AI-based games might be easily abused or misused. Going across Web of Science, and performing bibliometric analyses using VOSviewer, and qualitative analysis using CitNetExplorer, the authors identify the top 10 scientists, organizations, and countries conducting research in this area. The authors pointed out three AI ethical dimensions in the education field: deontology, ethics as a norm based on responsibilities; utilitarianism, benefits of ethics for the individual and the society; and virtue, ethics as oriented to reach goals and objectives. They also individuate the following main principles of AI ethics in education: transparency, responsibility, privacy, justice, fairness, equity, responsibility and no maleficience. Besides the ethical aspects of AI education, when focusing on learning and acculturation processes, one crucial aspect deserving attention concerns the modalities through which contents are transmitted to others. A significant challenge for modern society is to efficiently communicate meanings, especially difficult ones, which usually consist of values, numbers and quantities.

Winter and Marghetis invite us to think about how numerical meaning emerges holistically in multimodal messages. They start from the assumption that numerical communication is typically investigated by focusing on specific modalities of communication, such as speech, writing, signs, gestures, and graphs. Capitalizing on this premise, the authors make a case for the quintessential multimodal nature of numerical communication, arguing that interactions among modalities play a crucial role in the way this capability is conceptualized. On the one hand, different modalities share commonalities, including shared cognitive mappings and semiotic principles, as well as the capability to focus on either exact or approximate expressions. On the other hand, there are also important differences as the timescale: sequential vs. simultaneous presentation, permanence, and the distinct reliance on expertise among different modalities. In conclusion, multimodality is not additive.

Finally, a longstanding question that is still far from being addressed in the humanities field, is faced in Ruan's manuscript. The author deals with the problem of reconciling the scientific, especially the neuroscientific results with the subjective experience of reality: the so-called “hard problem of consciousness” originally identified by David Chalmers, that is the unbridgeable gap between the first-person perspective and the third-person perspective.

The author's effort is conveyed to shorten the gap between philosophical theories and neuroscientific approaches to consciousness. He provides a criterion based on necessary and sufficient conditions that could provide an empirical version of the “hard problem” and that could be used in the evaluation of several existing neuroscientific theories of consciousness.

In conclusion, this Research Topic offers new and diversified insights about (i) the theoretical background pertaining to the relationship between mind and body, ranging from epistemological perspectives to the role of embodiment in human creativity, (ii) the integration of Artificial Intelligence in psychological research and the related ethical aspects in the education field, (iii) the importance of multimodality in numerical communication, and the necessity (iv) to deeply reflect on the extent to which scientific results can account for the subjective experience of reality.

## Author contributions

CF: Writing—review and editing. FC: Writing—review and editing. AB: Writing—original draft.
